# The Rôle of the "Liver."

**Published:** 1900-04-21

**Authors:** 


### THE RÔLE OF THE "LIVER."

Writing about the influence of the liver upon health Mr. Hutchinson[1] says that the popular creed is not a mistake, and that half our miseries are due to functional disorders of the liver. The expressions "biliousness," "liver out of order," "sluggish liver," and the like are, he says, perfectly well founded, and it is our duty not to pass such things over in the complacency of superior knowledge, but patiently to inquire as to their real nature. First we have to recognise that the functional activity of the liver is most unquestionably liable to be influenced by causes acting through the nervous system, while this disturbed function may again become the excitant of other reflex functional disturbances, more especially of the vaso motor system, causing, for example, inequalities of circulation, cold extremities, pale face, and low spirits. In addition to all this an accumulation takes place in the blood of ingredients which ought to have been eliminated, and the intestinal contents are deprived of elements essential to healthy digestion, and thus, as well as reflex effects, a definite toxic influence is brought into play. What, then, are the symptoms of this disturbance of hepatic function? A yellow skin, clay-coloured fæces, high-coloured urine, and almost invariably constipation. But there are mental symptoms also. "The witty reply to the absurd question, 'Is life worth living?' 'That depends upon the liver,' was really . . . scarcely an exaggeration." Ill temper, melancholy, many suicides, cruelties without end, and not a few murders, are to be charged to disorders of the liver, while "a 'sunny disposition' is probably in most instances equivalent for a 'well-ordered liver.'" The liver must not then be regarded as a part of the system which can be isolated. It is in close organic sympathy with other organs, and, more especially during the period which intervenes between puberty and the grand climacteric, it is in very close relation with the sexual organs, so that the results of disturbances of these two systems are often inextricably intermingled. It is well to remember, then, that in many cases, symptoms attributed to derangement of the sexual apparatus have the liver as a most important link in the chain of their causation, and, indeed, that it may be only through the disturbance of its function that the symptoms complained of may have been brought into existence. "Whilst freely admitting that we must take the whole body into our consideration, the foremost importance of this viscus must yet be re-asserted." As is well known, Mr. Hutchinson has much confidence in the beneficial effects of mercury in many conditions, in the treatment of which some of the younger generation either adopt "milder" measures, or send their patients to drink strong waters under the superintendence of German physicians. Speaking of the use of long courses of mercury in some "liver" cases, he says: "I have often known patients who had been under long mercurial treatment for syphilis remark exultingly at the end of it, 'I have quite got rid of my headaches, which used to be the torment of my life.' Surely we may learn a lesson from such facts. It is not necessary that a person should have syphilis in order to secure the advantage of a year's course of mercury."

[1] Archives of Surgery, Jan., 1900.

